# Decreased ovarian reserve, dysregulation of mitochondrial biogenesis, and increased lipid peroxidation in female mouse offspring exposed to an obesogenic maternal diet

**DOI:** 10.1096/fj.15-280800

**Published:** 2015-12-23

**Authors:** Catherine E. Aiken, Jane L. Tarry-Adkins, Naomi C. Penfold, Laura Dearden, Susan E. Ozanne

**Affiliations:** *University of Cambridge Metabolic Research Laboratories and MRC Metabolic Diseases Unit, Institute of Metabolic Science, Addenbrooke's Hospital, Cambridge, United Kingdom; and ^†^Department of Obstetrics and Gynaecology, University of Cambridge, The Rosie Hospital and National Institute for Health Research Cambridge Comprehensive Biomedical Research Centre, Cambridge, United Kingdom

**Keywords:** developmental programming, reproductive potential, primordial follicle, lipoxygenase

## Abstract

Maternal diet during pregnancy influences the later life reproductive potential of female offspring. We investigate the molecular mechanisms underlying the depletion of ovarian follicular reserve in young adult females following exposure to obesogenic diet in early life. Furthermore, we explore the interaction between adverse maternal diet and postweaning diet in generating reduced ovarian reserve. Female mice were exposed to either maternal obesogenic (high fat/high sugar) or maternal control diet *in utero* and during lactation, then weaned onto either obesogenic or control diet. At 12 wk of age, the offspring ovarian reserve was depleted following exposure to maternal obesogenic diet (*P* < 0.05), but not postweaning obesogenic diet. Maternal obesogenic diet was associated with increased mitochondrial DNA biogenesis (copy number *P* < 0.05; transcription factor A, mitochondrial expression *P* < 0.05), increased mitochondrial antioxidant defenses [manganese superoxide dismutase (*MnSOD*) *P* < 0.05; copper/zinc superoxide dismutase *P* < 0.05; glutathione peroxidase 4 *P* < 0.01] and increased lipoxygenase expression (arachidonate 12-lipoxygenase *P* < 0.05; arachidonate 15-lipoxygenase *P* < 0.05) in the ovary. There was also significantly increased expression of the transcriptional regulator NF-κB (*P* < 0.05). There was no effect of postweaning diet on any measured ovarian parameters. Maternal diet thus plays a central role in determining follicular reserve in adult female offspring. Our observations suggest that lipid peroxidation and mitochondrial biogenesis are the key intracellular pathways involved in programming of ovarian reserve.—Aiken, C. E., Tarry-Adkins, J. L., Penfold, N. C., Dearden, L., Ozanne, S. E. Decreased ovarian reserve, dysregulation of mitochondrial biogenesis, and increased lipid peroxidation in female mouse offspring exposed to an obesogenic maternal diet.

It is well established that the early life environment plays an important role in programming many aspects of physiology and metabolism in the developing organism. Developmental programming persists throughout life and may be transmitted to subsequent generations (1, 2). Suboptimal maternal nutrition prior to and during pregnancy is a major mechanism through which the developing conceptus can acquire maladaptive programming. In developed countries, by far the most common suboptimal maternal diet is a combination of high sugar and high fat consumption, which leads to obesity. In women of childbearing age, obesity already affects at least 15% of the United Kingdom population (3). Globally, the rates of maternal obesity continue to rise (4, 5), leading to higher risks of immediate (6) and adverse outcomes for both mother and her offspring later in life (7).

The reproductive system appears to be exquisitely sensitive to early life influences. Maternal diet during pregnancy affects numerous parameters of offspring reproductive function (8) including follicular reserve (9), ovarian vascularity (10), and estrous cycling (11) in rat models utilizing a variety of adverse nutritional stimuli. Although reproductive phenotypes have been previously characterized in various developmental programming models, the underlying molecular mechanisms remain unclear. In other organ systems, accumulation of oxidative stress (12), deficits of key antioxidants (13), and inflammation (14) have been observed in response to suboptimal early life environments.

The interplay between maternal diet during pregnancy and postweaning offspring diet in generating adverse programming effects has been investigated by numerous studies (15–17). An obesogenic postnatal diet may exaggerate the effects of developmental programming by maternal diet (18), or conversely postnatal interventions such as antioxidant therapy (13) or growth hormone treatment (19) may help to prevent the development of an adverse phenotype later in life.

In this study, we utilize a mouse model of maternal diet-induced obesity using an obesogenic diet that is rich in simple sugars and saturated fat to better understand the effects of a typical Western diet during pregnancy on reproductive outcomes in female offspring. The primary aim of this study is to determine which, if any, of the numerous intracellular pathways previously implicated in developmental programming are involved in generating an adverse phenotype in the ovary. The second aim of this study is to examine the combined effects of adverse maternal and offspring diets on ovarian reserve.

## MATERIALS AND METHODS

### Animal work

All animal experiments underwent ethical review by the University of Cambridge Animal Welfare and Ethical Review Board and were carried out under the UK Home Office Animals (Scientific Procedures) Act (1986, United Kingdom). Female C57BL/6J mice were randomized to be fed *ad libitum* either a standard laboratory chow diet (7% simple sugars/3% fat; Special Dietary Services, Witham, United Kingdom) or an obesogenic diet (10% simple sugars/20% animal lard; Special Diets Services). The obesogenic diet was supplemented with a separate pot of sweetened condensed milk (55% simple sugars/8% fat; Nestle United Kingdom, Gatwick, United Kingdom) available to the animals within the cage. A detailed description of the dietary regimen has been published previously (20). The female mice were placed on the allocated diet 6 wk prior to first mating. The first was discarded after weaning, and only proven-breeder females were used for the experimental protocols. Second matings occurred when the females on the obesogenic diet had reached at least 10 g absolute fat mass, as assessed by time domain nuclear resonance imaging (Minispec Time Domain Nuclear Resonance; Bruker Optics, Billerica, MA, USA). The female mice remained on their allocated diets throughout the breeding, pregnancy, and lactation phases. After delivery, each litter was culled to 6 pups at random to standardize their plane of nutrition from postnatal day 3 in all litters. There was no significant difference in the preculling litter size between obesogenic and control litters. Equal sex ratios within the litters were maintained as far as possible. After weaning at day 21, female offspring were randomly allocated to either the control or the obesogenic diets (identical to those used for the dams) and remained on these diets for the duration of the study. Body weight and food intake were measured weekly. At 12 wk of age, offspring total and fat mass were assessed by weighing and by time domain nuclear resonance imaging (Bruker Optics), respectively. Following overnight food withdrawal, the female offspring were weighed and then culled by CO_2_ asphyxiation and cervical dislocation. Ovaries were dissected and weighed immediately. One ovary from each animal was snap-frozen in liquid nitrogen or dry ice, and stored at −80°C, the other was fixed in formalin/paraldehyde. The fixed ovary was sectioned and stained with hematoxylin and eosin to ensure equal distribution of estrous stages in each experimental group (data not shown).

### Primordial follicle quantification

Fixed ovaries were embedded and serially sectioned at 5 μm. Every third section was stained with hematoxylin and eosin for morphometric analysis (15 μm between analyzed sections, which should give an error rate of <10%) (21). Only follicles where the oocyte nucleus could be identified were counted to avoid repeat counts of the same follicle. Primordial follicles were identified morphologically as previously described (9). A single blinded observer assessed all slides to reduce heterogeneity in the analysis. Ovarian volume was calculated from cross-sectional area × slice thickness summed for each ovary, and the primordial follicle counts expressed as follicles per cubic millimeter of ovarian tissue.

### Gene expression analysis

An initial screen of 31 candidate genes was developed to test which molecular pathways might be involved in generating adverse reproductive system effects (**Table 1**). These genes were chosen based on *1*) previous work on reproductive programming (9–11, 22), *2*) knowledge of programming mechanisms in other organ systems (12, 13, 23), and *3*) relevant literature review. The genes forming the initial screen are listed with their *q* values in **Table 2**. All other gene expression levels tested were as a result of prespecified hypotheses, based on the initial screen results. Expression levels were measured in whole snap-frozen ovaries.

**TABLE 1.** Primer sequences for gene expression studies

Primer	Sequence (F)	Sequence (R)	Product size (bp)
*Tfam*	AGCTGAGTGGAAAGCATACAAA	CCTTCTCCATACCCATCAGC	83
*Nrf2*	AGCAAGACTTGGGCCACTTA	CTGAGCCGCCTTTTCAGTAG	97
*Pgc-1α*	TTACACCTGTGACGCTTTCG	TTGCTTCCGTCCACAAAAGT	97
*MnSOD*	GTGTGGGAGCACGCTTACTA	TCTCCCAGTTGATTACATTCCA	85
*CuZnSOD*	AGATGACTTGGGCAAAGGTG	AATCCCAATCACTCCACAGG	85
*Ccs*	CCTTTTCCAGAACCCCAAG	CTCGCTCCTCCCAGATAGTG	66
*ECSOD*	ATCCACGTGCATGAGTTCG	ACCTCCATCGGGTTGTAGTG	74
*Catalase*	CCCCCAACTATTACCCCAAC	TGAAGCGTTTCACATCTACAGC	96
*Gpx1*	ACCCGCTCTTTACCTTCCTG	ACACCGGAGACCAAATGATG	99
*Gpx4*	CCGGCTACAACGTCAAGTTT	CCTTGGGCTGGACTTTCAT	96
*Gr*	GGGATTGGCTGTGATGAGAT	GAATGGCAACTGTGTTGTCG	91
*Hmox1*	AGACACCCCGAGGGAAAC	GAGAGTGAGGACCCACTGGA	74
*Prdx1*	CACGGAGATCATTGCTTTCA	CCAATCACTTGGCAGTTGAG	66
*Prdx3*	TCGTCAAGCACCTGAGTGTC	CTCCATGGGTCTCCACAAAC	99
*Txnrd1*	CAAACGCTGGAGAGGTGAC	TGTCCAGCTGCTGCTTAGTC	72
*Nox4*	TTGATGGTCCATTTGGAAGC	CCTCCAGCCACACAGAGACT	70
*Xo*	TAACGCCAAACAGCTCTTCC	CAAGCGTTTCGGATCTTCTC	63
*Nf-kb*	GCTACACAGAGGCCATTGAAG	AGAGGCAGACAGTGGACCTG	100
*Alox12*	CCAGCTCCAACACTGTTCCT	AGCTCCTGCAGTTGGAAATC	95
*Alox15*	CCAGCATTCAGAGGAACACTT	CAGCCAGCTCCTCTCTGAAC	74
*Ppar-γ*	ACAGACCTCAGGCAGATCGT	GGGTGAAGGCTCATGTCTGT	83
*Ltb4r1*	GGCTTCGTGGTCAAGCTACT	GACAGGCAGGTGTGTCCTTC	100
*Ptgs2*	GGAGCACCATTCTCCTTGAA	TAAAACCCACTTCGCCTCCA	94
*p53*	TTTGAGGTTCGTGTTTGTGC	CCTTTTTGCGGAAATTTTCTT	70
*p21*	AGGCCCAGTACTTCCTCTGC	GCTCAGACACCAGAGTGCAA	88
*p16^INK^*	GACACGCTGGTGGTGCTG	TTGATGTCCCCGCTCTTG	99
*Ogg1*	GCTTAATGGCCCTTGACAAA	TTAGGATGCCAGCCGTAGTC	88
*Casp3*	ACGCGCACAAGCTAGAATTT	GAAGGACTCGAATTCCGTTG	74
*Col1A1*	CACTGCAAGAACAGCGTAGC	CCTCTGAGCTCGATCTCGTT	95
*IL6*	TCCTTCCTACCCCAATTTCC	GCCACTCCTTCTGTGACTCC	63
*IL1-β*	CAAGAGCAAAGTGGAGTTTGAG	CTTGTGCTCTGCTTGTGAGG	73
*Ccl2*	TGCCCTAAGGTCTTCAGCAC	TGTGGAAAAGGTAGTGGATGC	79
*Tnfα*	CGACTACGTGCTCCTCACC	ACGGCAGAGAGGAGGTTGAC	78
*Tgfβ1*	TGCCCTCTACAACCAACACA	CTTGCGACCCACGTAGTAGA	100
*Fth1*	TGAGTGAACAGGTGAAATCCA	TCTTGCGTAAGTTGGTCACG	60
*Myh6*	AGGCCAACACCAACCTGTC	CAGCTTGTTGACCTGGGACT	98
*Ppia*	CGGAGAGAAATTTGAGGATGA	TGTGTTTGGTCCAGCATTTG	85

*Ccl2*, chemokine (C-C motif) ligand 2; *ECSOD*, extracellular superoxide dismutase; F, forward; *Fth1*, ferritin heavy chain; *Gr*, glutathione reductase; *Gpx1*, glutathione peroxidase 1; *Gpx4*, glutathione peroxidase 4; *Hmox1*, hemoxygenase 1; *IL6*, interleukin 6; *IL1*-*β*, interleukin-1 β; *Ltb4r1*, leukotriene β 4 receptor 1; *Nox4*, NADPH oxidase 3; *Ogg1*, oxoguanine glycosylase 1; *Ppia*, peptidylprolyl isomerase A (cyclophilin A); *Prdx1*, peroxiredoxin 1; *Prdx3*, peroxiredoxin 3; *Ptgs2*, prostaglandin endoperoxide synthase 2; *p16^INK^*, cyclin dependent kinase inhibitor 2A; R, reverse; *Tgfβ1*, transforming growth factor β; *TNF-*α, tumor necrosis factor α; *Txnrd1*, thioredoxin; reductase 1; *Xo*, xanthine oxidase.

**TABLE 2.** q values (adjusted P values to correct for multiple comparison testing) for offspring diet and maternal diet effect in an initial screen of candidate gene expression

Gene	Offspring effect	Maternal effect
*Ppia*	0.97	0.93
*Ogg1*	0.97	0.29
*MnSOD*	0.97	0.09
*CuZnSOD*	0.74	0.06
*Ccs*	0.57	0.09
*ECSOD*	0.21	0.42
*Catalase*	0.19	0.99
*Nrf2*	0.68	0.06
*Gpx1*	0.41	0.78
*Gpx4*	0.74	<0.01
*GR*	0.31	0.43
*Hmox1*	0.31	0.09
*Prdx1*	0.68	0.49
*Prdx3*	0.37	0.10
*Txnd1*	0.60	0.21
*Xo*	0.31	0.42
*Nox4*	0.70	0.12
*Alox12*	0.41	0.06
*Cox2*	0.43	0.31
*NF-kb*	0.93	0.09
*IL6*	0.83	0.19
*IL1b*	0.16	0.31
*TnF-α*	0.19	0.10
*Tfam*	0.41	0.09
*Pgc-1α*	0.19	0.86
*Prc*	0.97	0.46
*p53*	0.41	0.19
*p21*	0.19	<0.01
*p16^INK^*	0.88	0.70
*Casp3*	0.60	0.29
*Col1A1*	0.39	0.31

*ECSOD*, extracellular superoxide dismutase; *Gr*, glutathione reductase; *Gpx1*, glutathione peroxidase 1; *Gpx4*, glutathione peroxidase 4; *Hmox1*, hemoxygenase 1; *IL6*, interleukin 6; *IL1-β*, interleukin-1 β; *Nox4*, NADPH oxidase 3; *Ogg1*, oxoguanine glycosylase 1; *Ppia*, peptidylprolyl isomerase A (cyclophilin A); *Prdx1*, peroxiredoxin 1; *Prdx3*, peroxiredoxin 3; *p16^INK^*, cyclin dependent kinase inhibitor 2A; *TNF-α*, tumor necrosis factor α; *Txnrd1*, thioredoxin; reductase 1; *Xo*, xanthine oxidase.

RNA was extracted using a miRNeasy mini Kit (Qiagen, Hilden, Germany). The kit was used according to the manufacturer’s instructions, with the addition of DNaseI digestion to ensure that the samples were free from genomic DNA contamination. The extracted RNA was quantified using a Nanodrop spectrophotometer (Nanodrop Technologies, Wilmington, DE, USA). cDNA was synthesized from 1 μg RNA using oligo-dT primers and M-MLV reverse transcriptase. Gene expression was quantified via RT-PCR (StepOne Plus machine; Applied Biosystems, Warrington, United Kingdom) using custom-designed primers (Sigma-Aldrich, Poole, United Kingdom; sequences in Table 2) and SYBR green reagents (Applied Biosystems). Equal efficiency of reverse transcription between all groups was confirmed using the housekeeper gene peptidylprolyl isomerase A (cyclophilin A), and absence of genomic DNA contamination was confirmed by quantifying myosin heavy chain 6 (*Myh6*), which was absent in all samples.

### Mitochondrial DNA copy number

Total DNA (mitochondrial and nuclear) was extracted using a DNeasy Blood and Tissue kit (Qiagen) according to the manufacturer’s instructions. A ratiometric assay of the levels of a single-copy mitochondrial gene, cytochrome c oxidase (*Cox1*), against a single-copy nuclear gene, nth endonuclease III-like 1 (*Nthl1*), was used to estimate the average copy number of mitochondrial DNA (mtDNA)/nuclear DNA (22) using PCR with SYBR green reagents (Applied Biosystems). Standard curves were created using 2-fold dilutions of standard mouse genomic DNA (Novagen, Darmstadt, Germany). The gradient of the standard curve (*y*) reflects the efficiency of the reaction, with an optimum value of 3.333 (100% efficiency). *C_t_* values for *Nthl1* were subtracted from those for *Cox1* to give Δ*C_t_*. A gradient of Δ*C_t_* across the dilution series < 0.1 was accepted. Average mtDNA copy number per nuclear genome (2 *Nthl1* copies) was calculated as 2 × 2^(Δ^*^Ct^*^)^.

### Statistical analysis

All data were initially analyzed using a 2-way ANOVA with maternal diet and offspring diet as the independent variables. To correct for multiple hypothesis testing of gene expression levels, *P* values were transformed to *q* values to take account of the false discovery rates using the p.adjust function in R stats package (R Foundation for Statistical Computing, Vienna, Austria). This adjustment was designed for this study to take account of the specific number of genes that were tested within the initial screen (24) and therefore to ensure that the *P* values were optimally transformed. Maternal diet effects were compared between groups using 2-tailed Student’s *t* tests. Data are represented as means ± sem. Where *P* values are reported, an α level <0.05 was considered statistically significant. All data analysis was conducted using the R statistical software package version 2.14.1 (R Foundation for Statistical Computing). In all cases, the number of litters = 8 for all groups. Study power was determined based on effect sizes for primordial follicle counts and mtDNA copy number observed in our previous studies of follicular reserve in other dietary models (2, 9).

## RESULTS

### Body weight and ovarian weights

**Figure 1.**
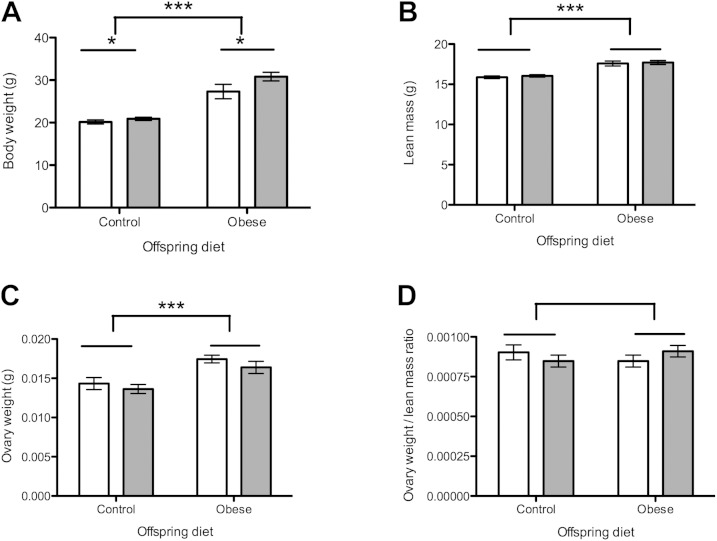
Body weight and ovarian weight at 12 wk of age. Open bars represent maternal control diet; gray bars represent maternal obesogenic diet. *A*) Total body weight in grams at 12 wk. *B*) Lean mass in grams at 12 wk. *C*) Absolute ovarian weight in grams at 12 wk. *D*) Ovarian weight adjusted for lean mass at 12 wk. *n* = 8 per group. *P < 0.05, ***P < 0.001.

At 12 wk of age, female offspring exposed to a postweaning obesogenic diet were significantly heavier than those eating a control diet (*P* < 0.001, **Fig. 1*A***). Females exposed to the obesogenic maternal diet were significantly heavier than those whose mothers ate the control diet during pregnancy (*P* < 0.05, Fig. 1*A*). These were independent effects, with no significant interaction. Lean mass at 12 wk of age was increased in offspring exposed to a postweaning obesogenic diet compared with controls (*P* < 0.001, Fig. 1*B*), but there was no effect of maternal diet on lean mass. Absolute ovarian weight was greater in offspring exposed to postweaning obesogenic diet (*P* < 0.001, Fig. 1*C*), with no effect of maternal diet. When ovarian weight was adjusted for lean mass at 12 wk, there was no significant difference in ovarian weight for any offspring or maternal dietary group (Fig. 1*D*).

### Primordial follicle counts

**Figure 2.**
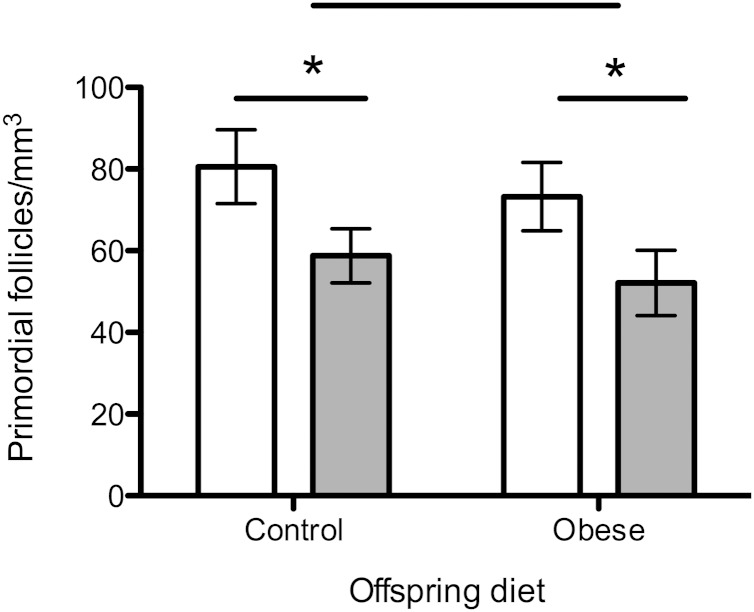
Primordial follicle counts of whole fixed ovaries at 12 wk of age. Open bars represent maternal control diet; gray bars represent maternal obesogenic diet. *n* = 8 per group. *P < 0.05.

Primordial follicle count per cubic millimeter of ovarian tissue was significantly reduced in 12-wk-old females exposed to the obesogenic maternal diet compared with controls (*P* < 0.05) (**Fig. 2**). There was no effect of postweaning offspring diet on primordial follicle counts.

### Ovarian mtDNA copy number

**Figure 3.**
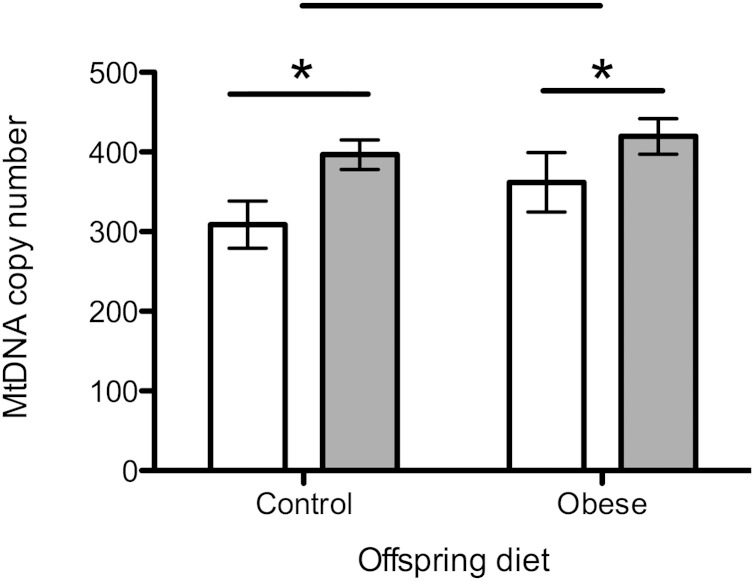
MtDNA copy number in the ovary. Open bars represent maternal control diet; gray bars represent maternal obesogenic diet. *n* = 8 per group. *P < 0.05.

There was an overall effect of maternal diet on mtDNA copy number, which was significantly higher in the maternal obesogenic diet group compared with the controls (*P* < 0.05) (**Fig. 3**). There was no significant effect of postweaning diet on mtDNA copy number in the ovary.

### Gene expression studies

An initial screen of 31 candidate genes was developed, and the screening results corrected for multiple hypothesis testing. After adjustment for multiple hypothesis testing, there were no significant effects of postweaning diet on expression levels of any of the candidate genes (Table 2). No effect of postweaning diet on ovarian phenotype at 12 wk of age was detected on any of the measured parameters within the study.

### Effect of maternal diet

A maternal obesogenic diet had a significant effect on ovarian primordial follicular reserve, mtDNA copy number, and expression levels of several candidate genes (Table 2), giving strong rationale to isolate the effects of maternal diet in further analyses. Based on these initial results, the expression levels of further genes were then tested on the same extracted samples based on prespecified hypotheses. Results of all gene expression studies are therefore presented only for control-fed offspring exposed to either maternal control or maternal obesogenic diet.

#### Pathways of mitochondrial biogenesis

**Figure 4.**
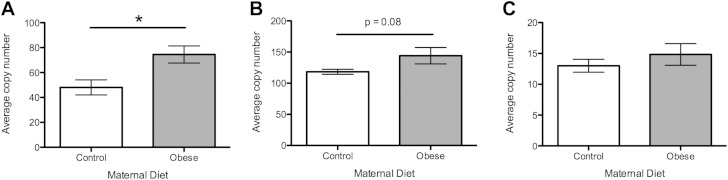
Expression of genes regulating mtDNA biogenesis. Open bars represent maternal control diet; gray bars represent maternal obesogenic diet. *A*) Expression of *Tfam*. *B*) Expression of *Nrf2*. *C*) Expression of *Pgc-1α*. *n* = 8 per group. *P < 0.05.

In view of the increased mtDNA copy number observed with the obesogenic maternal diet, levels of gene expression of the main regulators of mtDNA were assessed. Expression levels of mitochondrial transcription factor A (*Tfam*), the master regulator of mtDNA biogenesis, were significantly up-regulated by the maternal obesogenic diet compared with controls (**Fig. 4*A***; *P* < 0.05). There was also an increase in the expression of nuclear respiratory factor 2 (*Nrf2*), an important DNA-binding transcription factor that regulates *Tfam* and mtDNA biogenesis, but this did not reach statistical significance (Fig. 4*B*; *P* = 0.08). Peroxisome proliferator-activated receptor γ coactivator-1-α (*Pgc-1α*) was not significantly altered by maternal diet (Fig. 4*C*).

#### Mitochondrial antioxidant defense mechanisms

**Figure 5.**
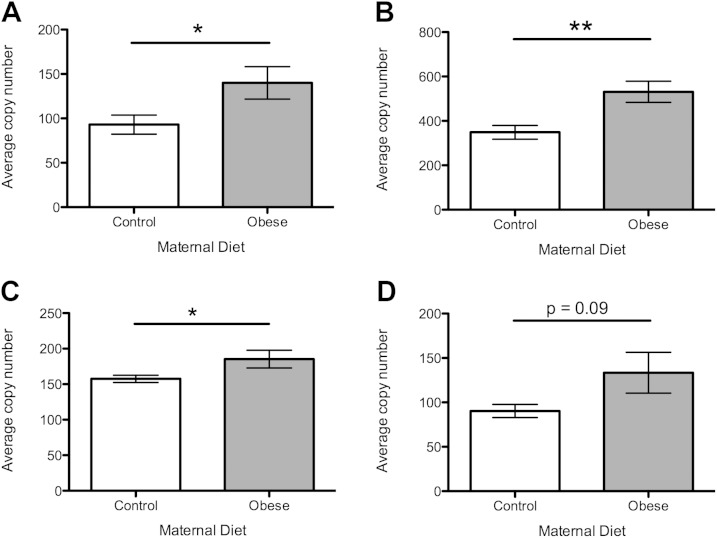
Expression of genes coding for mitochondrial anti-oxidant defenses. Open bars represent maternal control diet, gray bars represent maternal obesogenic diet. *A*) Expression of *MnSOD.* *B*) Expression of *Gpx4*. *C*) Expression of *CuZnSOD*. *D*) Expression of *Ccs. n* = 8 per group. *P < 0.05, **P < 0.01.

Expression levels of *MnSOD*, the major mechanism by which the superoxide anion is neutralized within the inner mitochondrial membrane, were significantly up-regulated in offspring exposed to a maternal obesogenic diet compared with control diet (**Fig. 5*A***; *P* < 0.05). Expression levels of glutathione peroxidase 4 (*Gpx4*), which in mice is spliced into a number of variants including a mitochondrial isoform (25), were higher in offspring exposed to maternal obesogenic diet (Fig. 5*B*; *P* < 0.01). Copper/zinc superoxide dismutase (*CuZnSOD*), which localizes to the mitochondrial intermembrane space as well as the cytoplasm, was significantly up-regulated with exposure to maternal obesogenic diet (Fig. 5*C*; *P* < 0.05). There was an increased expression of the copper chaperone (*Ccs*) of *CuZnSOD*, but this did not reach statistical significance (Fig. 5*D*; *P* = 0.09).

#### Oxidative stress response

**Figure 6.**
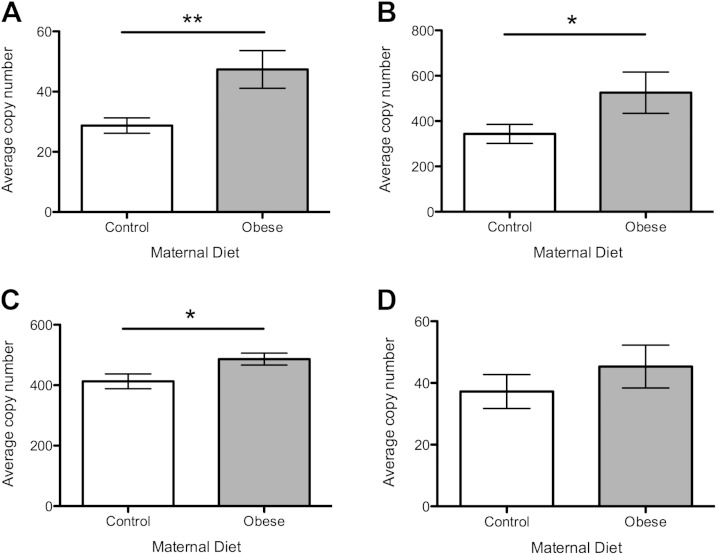
Expression of genes coding for oxidative stress related proteins. Open bars represent maternal control diet; gray bars represent maternal obesogenic diet. *A*) Expression of *Nf-kb*. *B*) Expression of *Alox12*. *C*) Expression of *Alox15*. *D*) Expression of *Ppar-γ. n* = 8 per group. *P < 0.05, **P < 0.01.

Exposure to the maternal obesogenic diet significantly increased ovarian expression of NF-κB DNA binding subunit (*Nf-kb*) (**Fig. 6*A***; *P* < 0.01). There was also significant up-regulation of both arachidonate 12-lipoxygenase (*Alox12*) and arachidonate 15-lipoxygenase (*Alox15*) expression in the maternal obesogenic diet group, reflecting increased lipid peroxidation (Fig. 6*B*, *C*; *P* < 0.05). There was no significant difference in the expression levels of peroxisome proliferator-activated receptor γ (*Ppar-γ*) between the 2 maternal diet groups (Fig. 6*D*).

#### Cellular ageing

None of the molecular markers of cellular ageing measured at the mRNA expression level analyzed in this study showed significant expression differences in response to maternal diet. Measured genes included *p53*, *p21*, *p16^Ink^*, caspase-3 (*Casp3*), and collagenase 1-α 1 (*Col1A1*) (data not shown).

## DISCUSSION

**Figure 7.**
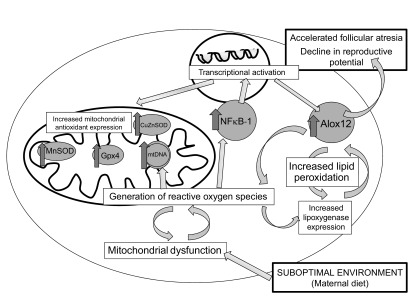
Schematic diagram of proposed effects of suboptimal maternal diet on intracellular pathways in the ovary.

Generation of an adverse phenotype in the reproductive system of young female offspring after exposure to a suboptimal maternal diet has been shown in a number of animal models (9–11) and human cohorts (8, 26, 27). This study advances our understanding of how programming of ovarian reserve occurs by identifying mitochondrial biogenesis and lipid peroxidation as the key mechanisms that are altered in the ovary after exposure to a maternal, but not postweaning, obesogenic diet (summarized in **Fig. 7**).

We show that in young adult females, a postweaning obesogenic diet does not impact on follicular reserve, mtDNA copy number, or any of the measured parameters reflecting ovarian stress generation. It is possible that the young adults studied here might go on to develop a phenotype of accelerated follicular atresia later in their reproductive life spans with continued exposure to obesogenic diet, which has been shown to have a detrimental effect on other metabolically active tissues (28). However, we show that they were unimpaired at the early time studied and that exposure to an obesogenic diet early in postweaning life does not reduce the initial pool of primordial follicles even though it impacts on body weight and body composition. These findings place the intrauterine environment clearly in the center of determining reproductive potential in adulthood, with the potential to impact on how reproductive capacity declines in later life. Although we show here that an early postweaning obesogenic diet does not directly affect follicular reserve, other studies have indicated that there is nonetheless some effect of postnatal high-fat diet on steroid-synthesis related parameters of reproductive function including pubertal age (11) and estrous cycling (22) in rat offspring. Follicular reserve is likely to differ from the somatic steroid-dependent aspects of reproductive function 1) because the generation of the primordial follicular pool occurs exclusively in late prenatal and early postnatal life and 2) because of the complex interplay between folliculogenesis and ovarian cytokines, which are likely to be influenced *in utero* by maternal diet (29). Hence, the early life environment would be expected to be the prime determinant of ovarian follicular reserve prior to significant follicular atresia with estrous cycling, which is in keeping with our findings.

We observed significant effects of a maternal obesogenic diet on mitochondrial biogenesis in the ovaries of young female offspring. The observed increase in mtDNA copy number, combined with increased gene expression of the main regulators of mtDNA biogenesis (*Tfam* and *Pgc-1α*), implies a compensated deficit in mitochondrial function. Mitochondria occupy a unique and critical position in the intracellular redox balance, in that they are both the primary generators of oxidative stress, yet particularly vulnerable to its effects (30, 31). An increase in mtDNA biogenesis early in life is therefore often a useful compensation for inefficient ATP production but leaves the cell with an increased lifetime exposure to reactive oxygen species. Increased lifetime exposure to such species can then lead to further cellular damage and eventually a positive feedback cycle of early decompensation later in life. In the young adult offspring studied here, there is also evidence of up-regulation of the major mitochondrial antioxidant defense mechanisms, in particular *MnSOD* and *Gpx4*, in keeping with the need for increased protection from reactive oxygen species.

In addition to dysregulation of mitochondrial biogenesis, we also observed an increase in the key enzymes involved in lipid peroxidation: *Alox12* and *Alox15*. Lipid peroxidation is implicated in the pathogenesis of many oxidative stress-related disease states, including ageing and metabolic diseases (24). Understanding of the multiple roles of lipoxygenases (other than in the classic arachidonic acid cascade) is increasing (32), including a clearer picture of their role in generating the cellular redox balance. Lipoxygenases are pro-oxidative enzymes: by forming hydroperoxy lipids, they can alter the redox state and gene expression pattern within the cell overall (33). Aside from the implications for cellular redox balance, an increase in lipoxygenase activity also leads to greater oxidation of membrane lipids, which in turn impairs the normal functions of the cell membrane and membrane-bound enzymes (34). Such impairment at the tissue level early in reproductive life may lead to increasing dysregulation of normal ovarian function later in life. Furthermore, there may be direct effects of *Alox12* expression on both ovulation and follicular reserve: *Alox12* is expressed in granulosa cells, thecal cells, and follicular fluid at the time of ovulation in the rat (35, 36), and inhibition of lipoxygenase can impair ovulation (36). Intriguingly, polymorphisms in the *Alox12* gene have been linked with early age at natural menopause in various human populations (37, 38), implying that their expression may be key to follicular reserve later in life.

Our observations of increased levels of intracellular redox regulators are strengthened by the associated up-regulation of downstream transcriptional pathways. In particular, we observed that the central transcriptional regulator *Nf-kb* was significantly up-regulated in the ovaries of offspring exposed to an obesogenic maternal diet, even at a young age. *Nf-kb* expression is induced in response to increased levels of reactive oxygen species (39) and has been implicated in a wide range of pathologic processes including inflammation and ageing (40). In the ovary, *Nf-kb* plays a role in regulating granulosa cell development during the formation of ovarian follicles (41, 42), which may explain the association observed in our study between maternal obesity and reduced primordial follicular reserve. Furthermore, up-regulation of *Nf-kb* expression has previously been demonstrated in the ovaries of young obese female mice prior to the onset of ovarian dysfunction (43). The response to a suboptimal early life environment in other organ systems also involves increased expression of *Nf-kb* (44–46).

We have previously observed that the end products of lipid peroxidation are significantly increased in the ovary in older rat offspring exposed to a maternal low-protein diet (9). Unfortunately, due to the very small absolute amount of ovarian tissue present in the mouse at this relatively young age, it was not possible to directly measure the end products of lipid peroxidation in this model system in addition to performing histologic, DNA, and extensive gene expression studies.

Our conclusion that a maternal obesogenic diet reduces ovarian follicular reserve, dysregulates ovarian mitochondrial biogenesis, and increases lipid peroxidation in young female offspring has important implications for maintaining reproductive potential at a population level, particularly in light of trends in many populations toward increasing maternal obesity (4, 5). Important future directions for this work include identifying possible interventions for primary or secondary prevention of adverse reproductive phenotypes in female offspring of obese mothers. Several interventions have previously been demonstrated to be potentially effective in protecting against programmed phenotypes when applied either preconception (47) or during pregnancy (48). Protection from adverse programming is not as well explored in the reproductive system; however, supplementation of maternal high-fat diet with conjugated linoleic acid has previously been shown to prevent precocious puberty in female offspring (49). However, it is not always possible to identify and intervene in high-risk pregnancies in time to prevent adverse phenotypes developing, and thus there is a need to develop parallel interventions that can be applied postnatally. Recent data suggest postnatal dietary supplementation with the powerful endogenous antioxidant coenzyme Q_10_ at physiologic doses (50) as a candidate intervention with good prospects for translation to human cohorts.

## Abbreviations

*Alox12*: arachidonate 12-lipoxygenase
*Alox15*: arachidonate 15-lipoxygenase
*Casp3*: caspase-3
*Ccs*: copper chaperone for superoxide dismutase
*Col1A1*: collagenase 1-α 1
*Cox1*: cytochrome c oxidase
*CuZnSOD*: copper/zinc superoxide dismutase
*Gpx4*: glutathione peroxidase 4
*MnSOD*: manganese superoxide dismutase
MtDNA: mitochondrial DNA
*Myh6*: myosin heavy chain 6
*Nf-kb*: nuclear factor κ-B DNA binding subunit
*Nrf2*: nuclear respiratory factor 2
*Nthl1*: nth endonuclease III-like 1
*Pgc-1α*: peroxisome proliferator-activated receptor γ coactivator-1-α
*Ppar-γ*: peroxisome proliferator-activated receptor γ
*Tfam*: mitochondrial transcription factor A
